# Correlation of Neurodegenerative Biomarkers and Functional Outcome in Patients with Relapsing–Remitting Multiple Sclerosis

**DOI:** 10.3390/neurolint17080123

**Published:** 2025-08-07

**Authors:** Elina Polunosika, Monta Feldmane, Daina Pastare, Joel Simren, Kaj Blennow, Nauris Zdanovskis, Henrik Zetterberg, Renars Erts, Guntis Karelis

**Affiliations:** 1 Department of Neurology and Neurosurgery, Riga East University Hospital, LV-1038 Riga, Latvia; monta.feldmane.mf@gmail.com (M.F.); daina.pastare@gmail.com (D.P.); guntis.karelis@gmail.com (G.K.); 2 Department of Neurology and Neurosurgery, Rīga Stradiņš University, LV-1007 Riga, Latvia; 3 Department of Psychiatry and Neurochemistry, Institute of Neuroscience and Physiology, Sahlgrenska Academy, University of Gothenburg, 40530 Molndal, Sweden; joel.simren@gu.se (J.S.); kaj.blennow@neuro.gu.se (K.B.); henrik.zetterberg@clinchem.gu.se (H.Z.); 4 Clinical Neurochemistry Laboratory, Sahlgrenska University Hospital, 41345 Molndal, Sweden; 5 Department of Radiology, Riga East University Hospital, LV-1038 Riga, Latvia; nzdanovskis@gmail.com; 6 Department of Radiology, Rīga Stradiņš University, LV-1007 Riga, Latvia; 7 Department of Neurodegenerative Disease, UCL Institute of Neurology, London WC1N 3BG, UK; 8 UK Dementia Research Institute, University College London, London WC1E 6BT, UK; 9 Hong Kong Center for Neurodegenerative Diseases, Clear Water Bay, Hong Kong, China; 10 Wisconsin Alzheimer’s Disease Research Center, School of Medicine and Public Health, University of Wisconsin-Madison, Madison, WI 53792, USA; 11 Faculty of Medicine and Life Sciences, University of Latvia, LV-1050 Riga, Latvia; renars.erts@gmail.com; 12 Department of Infectology, Rīga Stradiņš University, LV-1007 Riga, Latvia

**Keywords:** relapsing–remitting multiple sclerosis, biomarkers, disability, neurodegeneration, neurofilament light chain, magnetic resonance imaging, Symbol Digit Modalities Test, Brief Visuospatial Memory Test-Revised

## Abstract

**Background and Objectives**: Multiple sclerosis (MS) is a chronic autoimmune, inflammatory, and neurodegenerative central nervous system disease. Neurodegeneration plays a central role in long-term disease progression. **Materials and Methods**: This cross-sectional study examined the relationship between neurodegenerative biomarkers, namely plasma neurofilament light chain (pNfL) levels and MRI-derived brain volume measurements, and clinical outcomes in 49 patients with relapsing–remitting multiple sclerosis (RRMS). Plasma NfL levels were quantified using Simoa technology, while MRI data was analyzed via FreeSurfer to measure volumes of grey and white matter, specific brain structures, and ventricular sizes. Cognitive performance was assessed using the Symbol Digit Modalities Test (SDMT) and Brief Visuospatial Memory Test-Revised (BVMT-R). Disability was evaluated using the Expanded Disability Status Scale (EDSS). **Results**: The results indicated significant positive correlations between SDMT scores and volumes of grey matter, white matter, and various subcortical structures, suggesting that preserved brain volume is linked to better cognitive performance. Negative correlations were observed between SDMT scores and ventricular volumes, as well as between SDMT scores and EDSS scores, implying that cognitive decline corresponds with structural brain deterioration and increased disability. No significant associations were found between BVMT-R scores and imaging data or disability measures. Plasma NfL levels showed significant correlations with early disease relapses and enlargement of the third and fourth ventricles, but not with brain volume, cognitive tests, or EDSS scores. **Conclusions**: These findings indicate that MRI-based brain volumetrics, particularly grey and white matter measures, are stronger indicators of cognitive function and disability in RRMS than plasma NfL.

## 1. Introduction

Multiple sclerosis (MS) is a chronic, neurodegenerative, immune-mediated disorder that predominantly affects young adults and often leads to progressive disability. Characterized by inflammation, demyelination, and neurodegeneration, MS manifests itself in various clinical subtypes—relapsing–remitting MS, primary progressive MS, secondary progressive MS, and clinically isolated syndrome, with relapsing–remitting MS (RRMS) being the most prevalent. RRMS frequently evolves into more disabling forms, accompanied by physical and cognitive decline [1].

Although inflammatory mechanisms are more prominent in the early stages of MS, neurodegeneration plays a central role in long-term disease progression and is now recognized as a characteristic present in all stages of the disease [2,3].

Consequently, identifying sensitive and reliable biomarkers of neurodegeneration is crucial to understanding MS pathogenesis and predicting clinical outcomes. Biomarkers in MS can be broadly categorized into diagnostic, prognostic, disease activity, and treatment-response indicators [4,5]; these include both inflammatory and neurodegenerative biomarkers [6]. This study focused on neurodegenerative biomarkers in multiple sclerosis—namely, plasma neurofilament light chain (pNfL) levels and magnetic resonance imaging (MRI)-based brain volume measurements.

Neurofilament light chain (NfL), a structural protein of the neuronal cytoskeleton, is a well-established biomarker of neuroaxonal injury and has emerged as a valuable non-invasive biomarker for monitoring disease activity and progression. Elevated levels of NfL in cerebrospinal fluid and blood reflect active neurodegeneration and have been shown to correlate with brain atrophy, making it a valuable indicator of cognitive decline, especially in the early stages of MS [7], disease activity, progression, and treatment response in multiple sclerosis (MS) [8,9]. Although NfL is released primarily into cerebrospinal fluid, small amounts also enter the bloodstream, where they can be reliably measured using advanced techniques such as single molecule array (Simoa) assays [8]. Blood NfL has shown utility not only in MS but also in other neurological conditions such as traumatic brain injury, acute stroke, HIV encephalopathy, and neurodegenerative disorders [8,10,11]. NfL levels can also be affected by body mass index (BMI) [12,13,14,15,16]. Although some studies report a positive correlation between NfL levels and age, the findings regarding the influence of gender and age on NfL concentrations remain inconsistent [7]. Similarly, advanced MRI techniques allow for detailed evaluation of brain structural changes, such as cortical grey matter and white matter atrophy, which are strongly associated with cognitive impairment and physical disability [17]. Despite the recognized value of these individual biomarkers, the relationship between neuroimaging findings, blood-based biomarkers, and clinical outcomes remains incompletely understood.

The aim of this cross-sectional study was to examine the associations between neurodegenerative biomarkers—specifically plasma neurofilament light chain (pNfL) levels and MRI-derived brain volume measurements—and clinical outcomes, including cognitive performance and physical disability, in patients with early-stage relapsing–remitting multiple sclerosis (RRMS) at a young age. For the evaluation of cognitive functions, we used the Symbol Digit Modalities Test (SDMT) and the Brief Visuospatial Memory Test-Revised (BVMT-R). The SDMT is a widely used neuropsychological tool to assess cognitive processing speed, attention, and working memory. Lower scores are associated with greater disability, brain atrophy, and lesion burden in MS, making the SDMT a reliable and efficient tool for both clinical and research settings [18]. The BVMT-R assesses visuospatial learning and memory—functions often affected in MS [19]. The BVMT-R has been shown to correlate with disease progression in MS, regardless of age or level of education [20]. While SDMT is more sensitive to early cognitive changes, particularly those related to processing speed, BVMT-R provides valuable information about memory impairment. Together, these tests offer a comprehensive cognitive profile and are frequently used in MS research to assess the impact of the disease and monitor progression [21,22,23]. Their combined use, alongside MRI and biochemical markers such as pNfL, enhances the assessment of cognitive function and disease progression in RRMS patients. The study sought to determine the relative strength of these biomarkers in reflecting disease burden and functional status in RRMS.

## 2. Materials and Methods

This cross-sectional study was conducted at Riga East University Hospital and included 49 patients diagnosed with relapsing–remitting multiple sclerosis (RRMS). The objective of this study was to evaluate the relationship between plasma neurofilament light chain (pNfL) levels and MRI-based brain volumetric measures with cognitive function and disability in individuals with relapsing–remitting multiple sclerosis (RRMS). Specifically, the study aimed to determine how well these biomarkers reflect neurodegeneration and predict clinical outcomes such as cognitive performance, assessed by SDMT and BVMT-R, and physical disability, measured by the EDSS. Plasma neurofilament light chain (pNfL) levels were measured as a biomarker of neuroaxonal damage. Structural brain changes were assessed using magnetic resonance imaging (MRI), focusing on cortical grey matter volume, cerebral white matter volume, subcortical brain structures, and the dimensions of the ventricles.

Clinical and demographic data were collected, including age, sex, education level, disease duration, the number of relapses within the first five years, and disability status measured by the Expanded Disability Status Scale (EDSS). Cognitive function was assessed using two standardized neuropsychological tests: the Symbol Digit Modalities Test (SDMT) and the Brief Visuospatial Memory Test-Revised (BVMT-R).

Inclusion criteria were patients with multiple sclerosis who had a relapsing–remitting disease course based on the McDonald criteria (2017) [24], a disease duration of less than 20 years, and were receiving immunomodulating therapy, without specifying the exact medication administered. Participants were between 18 and 62 years of age, and had the ability to understand and perform the required tasks.

Exclusion criteria were patients with primary progressive or secondary progressive multiple sclerosis, and clinically isolated syndrome; patients with a disease duration of more than 20 years, and patients who were unable to understand and perform the required tasks. Additional exclusion criteria were a history of stroke, atrial fibrillation, myocardial infarction, chronic kidney disease, pregnancy, diabetes, neurodegenerative disease, and individuals who were obese (BMI > or = 30). Participants aged over 62 years were excluded.

During their visit, a functional neurological evaluation was performed using the EDSS, a tool designed to quantify disability in MS patients and track disease progression over time. The EDSS assesses eight functional systems—visual, pyramidal, cerebellar, brainstem, sensory, bowel and bladder, cerebral, and ambulation. Scores range from 0 to 10 in increments of 0.5. Scores between 0 and 5 indicate mild neurological impairment, with patients being able to walk without assistance. Scores between 6.0 and 8.5 reflect significant difficulties with walking and daily activities, while scores above 9.0 indicate that patients are bedridden. A score of 10 denotes death due to MS.

The SDMT is a widely used neuropsychological tool to assess cognitive processing speed, attention, and working memory. It is particularly sensitive in detecting cognitive impairment in MS, where processing speed deficits are among the most common early cognitive symptoms [25,26,27]. We used the written version of the SDMT, in which participants match symbols to corresponding numbers using a key presented at the top of the page. The task is timed (90 s), and the score reflects the number of correct responses.

The BVMT-R assesses visuospatial learning and memory—functions often affected in MS [19]. During the test, participants view six abstract geometric figures for 10 s and then attempt to reproduce them from memory. This process is repeated across three trials to assess learning, followed by a delayed recall trial after 25 min. An optional recognition component may also be administered. The scoring is based on the accuracy and spatial arrangement of the drawings [28].

In this study, plasma NfL (pNfL) levels were measured in blood samples collected from patients during morning clinical visits to reduce biological variability. Blood was drawn into 3 mL EDTA-K2 tubes and centrifuged at 2200× *g* for 10 min at 20 °C. Plasma was then transferred into 1.0 mL labeled aliquots using polypropylene pipettes and stored at −80 °C until analysis. The quantification of pNfL levels was performed using Simoa technology on the HD-X analyzer (Quanterix Corporation, Billerica, MA, USA), following the manufacturer’s standard NF-Light protocol [29]. Results were reported in picograms per milliliter (pg/mL).

All the study participants underwent structural magnetic resonance imaging (MRI) using at least a 1.5 Tesla MRI scanner, and 3D T1 (3D-T1 GRE) was used for brain segmentation. For brain segmentation, we used SynthSeg within the FreeSurfer environment, which applies a pretrained convolutional neural network to generate high-resolution labels of intracranial volume, cortical and subcortical grey matter, and white matter directly from bias-corrected, skull-stripped T1-weighted images. All outputs underwent visual quality control to exclude scans with motion or segmentation failures. The technical details of Freesurfer segmentation are described in prior publications [30].

Data were analyzed using statistical software R version 4.2.1 (R Core Team, 2022). Categorical variables were reported as counts and percentages and compared using Pearson’s chi-square test (for expected frequencies >5) or Fisher’s exact test (for frequencies <5). Odd ratios (ORs) were calculated for 2 × 2 tables. The normality of the continuous variables was assessed using the Shapiro–Wilk test. Normally distributed variables were presented as mean (M) ± standard deviation (SD), and non-normally distributed data as median (Md) with interquartile range (Q1–Q3). Student’s *t*-test was used to compare normally distributed variables between two groups; otherwise, the Mann–Whitney *U* test was applied. Pearson’s correlation coefficient (r) assessed relationships between normally distributed variables, with Spearman’s coefficient (rs) being used for non-normal data. Correlation strength was classified as weak (0.3–0.5), moderate (0.51–0.69), or strong (≥0.7). A 95% confidence interval (CI) was calculated for precision, and the *p*-value < 0.05 was considered statistically significant.

The study was carried out according to the Declaration of Helsinki and received approval from the Ethics Committee of the Ethics Board of Riga East University Hospital (No. 79/2018, 1 November 2018) and the Ethics Board of Rīga Stradiņš University (No. 6-3/120, 29 November 2018). The patients provided their written informed consent to participate in this study.

## 3. Results

### 3.1. General Characteristics

This study included 49 patients, with ages ranging from 30 to 57 years; 25 (51%) of the study participants were female, and 24 (49%) were male. The mean age among the study participants was 38.51 years (SD = 10.16), and the median age was 38 years [Q1 = 30; Q3 = 46.0]. The most frequently observed age was 30, with an age skewness coefficient of 0.05. Analysis of the age distribution revealed a deviation from normality (*p* = 0.04).

The median duration of the illness among the study participants was 8.00 years [Q1 = 6.00; Q3 = 10.0], with a minimum duration of two years and a maximum duration of 18 years.

On average, participants experienced three relapses within the first five years of the disease (Md = 3.00 [Q1 = 3.00; Q3 = 4.00]), the median EDSS score was 2.5 [Q1 = 2.00; Q3 = 3.50]. Among the participants, 19 participants (38.8%) had secondary education, including 11 females and 8 males; 30 participants (61.2%) had higher education, comprising 16 males and 14 females (Table 1).

### 3.2. Magnetic Resonance Imaging Data Analysis and Correlation with Disability

Importantly, in this study, EDSS was negatively correlated with multiple brain volume measures. The volumes of cortical grey matter in the left and right hemispheres and the total cortical grey matter showed weak to moderate inverse correlations (r = −0.32, 95% CI approximately [−0.56; −0.04]). The volumes of white matter in both hemispheres and the total volume of white matter were similarly associated (r ≈ −0.35 to −0.36), and the volume of subcortical grey matter was also negatively correlated with EDSS (r = −0.37, 95% CI [−0.59; −0.10]).

In addition to global brain volumes, EDSS was significantly associated with regional subcortical grey matter structures. Among these, thalamic atrophy showed the strongest correlation with disability, particularly in the left thalamus (r = −0.48, 95% CI [−0.67; −0.23]) and right thalamus (r = −0.45, 95% CI [−0.65; −0.19]). The right and left accumbens areas also showed moderate negative correlations (r = −0.40, 95% CI [−0.61; −0.13]), highlighting their relevance in EDSS progression. Moderate negative associations were observed with left pallidum (r = −0.35), left caudate (r = −0.29), and total grey matter volume (r = −0.31). Other regions like the hippocampus, amygdala, and putamen demonstrated weaker or non-significant trends, with confidence intervals crossing zero.

Conversely, ventricular enlargement was positively correlated with EDSS, suggesting compensatory expansion due to neurodegeneration. Notably, the fourth ventricle showed a moderate positive correlation (r = 0.44, 95% CI [0.18; 0.64]), and the third ventricle also correlated significantly (r = 0.28, 95% CI [0.00; 0.52]). Lateral ventricle volumes showed weaker associations (r = 0.21 to 0.27), with borderline statistical significance.

### 3.3. Cognitive Test Results

The median BVMT-R score was 3.0 [Q1 = 2.0; Q3 = 3.0], and the SDMT score was 82.3 [Q1 = 74.0; Q3 = 92.3], indicating moderate cognitive function within the cohort. We analysed the cognitive test results (SDMT and BVMTR) and the correlation between patient age, but we did not find any statistically significant correlation (*p* > 0.05), indicating that the worse cognitive test results were not associated with patient age in our study group.

**Figure 1:**
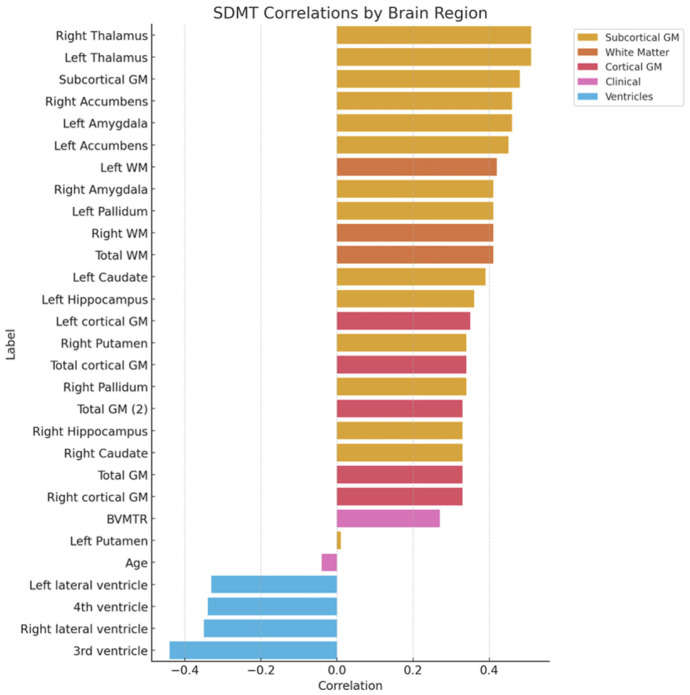
Correlation of SDMT and volumetrics of brain regions.

**Figure 2:**
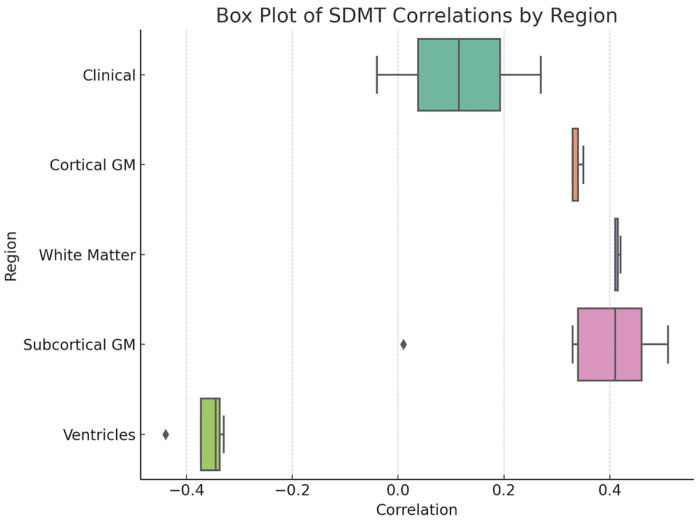
Correlation of SDMT results and volumetrics of brain regions.

**Figure 3:**
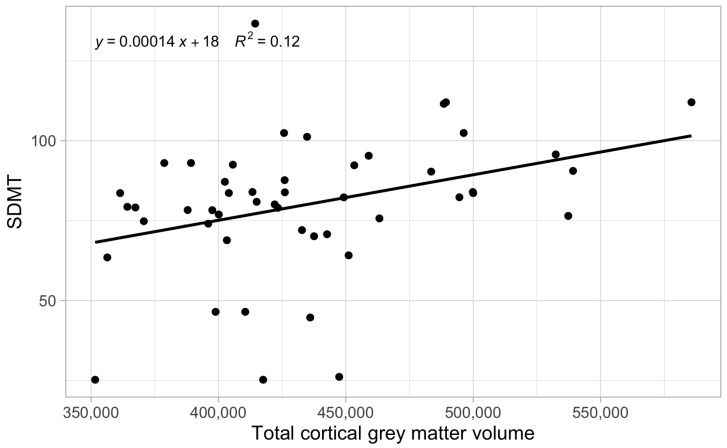
SDMT scores’ correlation with total cortical grey matter volume.

Our analysis revealed significant correlations between SDMT results and several volumetric measurements of the brain (Figure 1 and Figure 2). We observed positive correlations between SDMT scores and both the total volume of grey matter (r = 0.33, *p* < 0.05, 95% CI [0.05; 0.56]) and the total volume of white matter (r = 0.41, *p* < 0.01, 95% CI [0.15; 0.62]), indicating that larger volumes in these regions are associated with better cognitive performance (Figure 3). Furthermore, significant positive correlations were observed between SDMT scores and almost all brain structures measured, including the right and left accumbens area, amygdala, hippocampus, globus pallidus, nucleus caudatus, thalamus, and right putamen (all *p* < 0.05). However, no significant correlation was found between SDMT and the left putamen volume (r = 0.01, 95% CI [−0.27; 0.29]), indicating possible lateralized differences in structure–function relationships.

On the contrary, significant negative correlations were found between SDMT performance and ventricular volumes, including the third ventricle (r = −0.43, *p* = 0.01, 95% CI [−0.64; −0.18]), the fourth ventricle (r = −0.34, *p* = 0.01, 95% CI [−0.57; −0.07]), the left lateral ventricle (r = −0.33, *p* = 0.02, 95% CI [−0.56; −0.05]), and the right lateral ventricle (r = −0.35, *p* = 0.01, 95% CI [−0.58; −0.08]).

Our findings revealed a significant negative correlation between EDSS scores and the SDMT results (r = −0.55, *p* < 0.05, 95% CI [−0.72; −0.31]), but there was no significant correlation between EDSS and BVMT-R scores (r = −0.11, *p* > 0.05, 95% CI [−0.38; 0.17]).

**Figure 4:**
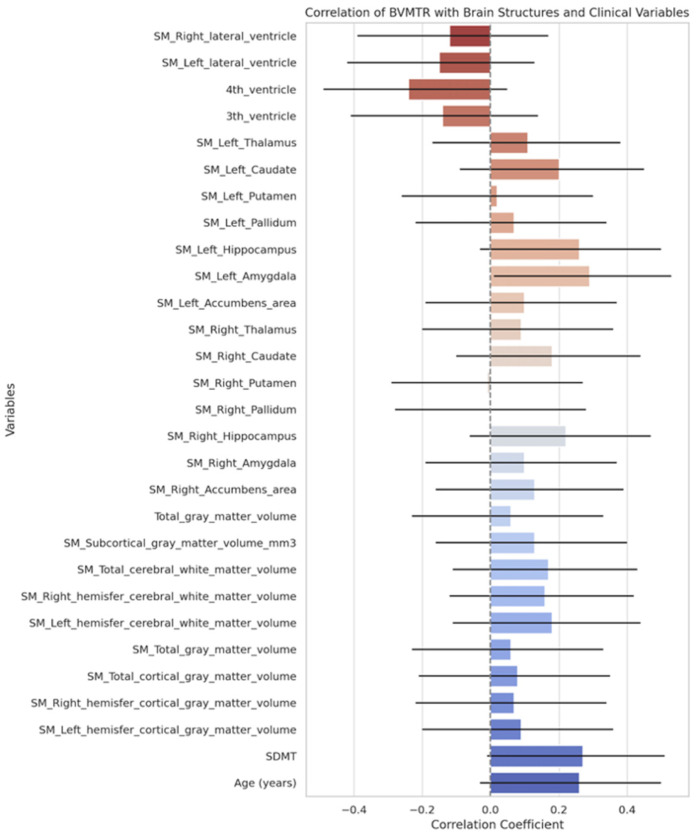
Correlation of BVMT-R results and volumetrics of brain regions and clinical variables.

Correlation analysis between BVMT-R scores and structural brain volumes revealed weak and statistically non-significant associations across nearly all regions (Figure 4).

### 3.4. Plasma Neurofilament Light Chain Levels

The median plasma neurofilament light chain (pNfL) level was 6.30 ng/L [4.60–10.9], suggesting varying degrees of ongoing neuroaxonal damage. pNfL showed modest correlations with age (r = 0.28, 95% CI [−0.01; 0.52]). Higher pNfL levels were associated with a greater number of relapses within the first five years of the disease (r = 0.3, *p* < 0.05, 95% CI [0.03; 0.54]). A weak positive correlation was also observed with EDSS (r = 0.20), though the confidence interval includes zero, indicating a trend but not a statistically robust association. Conversely, higher pNfL levels were weakly associated with lower SDMT scores (r = −0.24), suggesting a possible link between neuroaxonal injury and reduced processing speed, but not statistically significant. Correlations with BVMT-R, cortical and white matter volumes, and subcortical structures were all small (r < 0.15) and statistically nonsignificant, with wide confidence intervals crossing zero.

**Figure 5:**
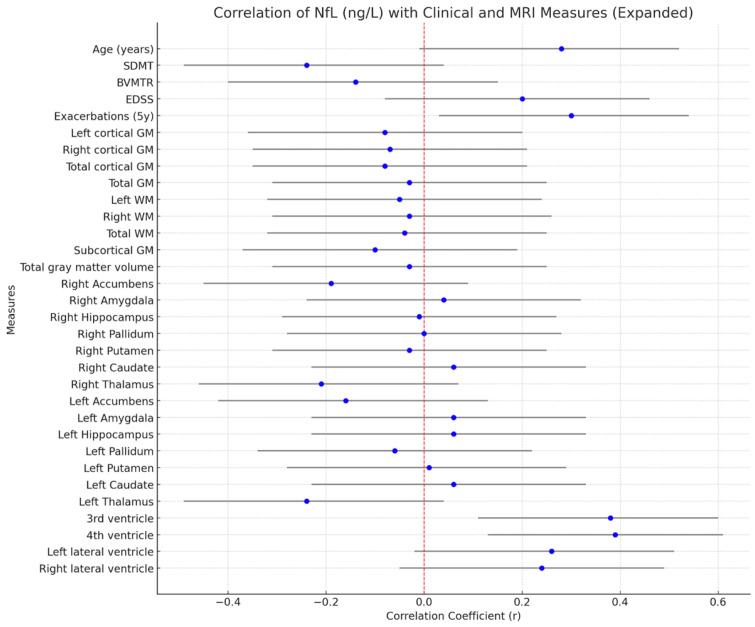
Correlation of pNfL (ng/L) with clinical and MRI measurements.

On the contrary, ventricular enlargement showed small to moderate positive correlations with pNfL: the third ventricle (r = 0.38, 95% CI [0.11; 0.60]) and the fourth ventricle (r = 0.39, 95% CI [0.13; 0.61]) showed the strongest positive associations. The correlations with lateral ventricles were weaker and nonsignificant (all *p* > 0.05). pNfL levels demonstrated no significant correlations with grey matter or subcortical volumes, with all correlation coefficients close to zero and 95% confidence intervals crossing zero (Figure 5).

## 4. Discussion

This cross-sectional study explored the relationships between plasma neurofilament light chain (pNfL) levels, brain volumetric measurements derived from magnetic resonance imaging, cognitive function, and clinical outcomes in patients with early-stage relapsing–remitting multiple sclerosis (RRMS). All correlation analyses were performed on 49 RRMS patients (*n* = 49). Volumetric MRI measurements stem from the growing recognition that neurodegeneration—manifested as brain atrophy—plays a central role in disease progression and cognitive decline in MS, often independent of inflammatory activity. While traditional clinical scales like the EDSS capture motor impairment, they often fail to detect subtle yet functionally important changes in cognition. Therefore, volumetric MRI offers an important complementary perspective by quantifying structural brain integrity. Primary findings indicate that structural brain changes, particularly grey and white matter volumes, are strongly associated with cognitive performance as evaluated by the Symbol Digit Modalities Test (SDMT), while pNfL levels showed limited correlations with cognitive and disability measures.

Our results align with previous research that shows that decreased grey and white matter volumes correlate with cognitive impairment in MS patients [26,31,32]. Significant positive correlations between SDMT scores and grey and white matter volumes, and multiple subcortical structures, emphasize the importance of structural preservation in maintaining cognitive function. In contrast, increased ventricular volumes, which likely reflect brain atrophy, were negatively correlated with SDMT scores, further underscoring the association between brain structural integrity and cognitive performance. Age was not significantly associated with SDMT in this study, indicating cognitive slowing may be more related to brain atrophy than age per se [33]. Also, it is proven that SDMT is the measure of choice for multiple sclerosis patients. The strong sensitivity of the SDMT in detecting cognitive impairment in MS may be attributed to its capacity to reflect the broad variability in cognitive performance among MS patients, despite its lack of specificity to a single cognitive domain, such as information processing speed [34]. SDMT test brevity, ease of administration, and strong psychometric properties further contribute to its practicality as a screening tool. However, due to its broad sensitivity, clinicians should interpret SDMT scores alongside more domain-specific assessments when a detailed cognitive profile is required. Future longitudinal studies may better clarify the temporal relationship between SDMT performance and regional atrophy.

Importantly, we did not find significant associations between the Brief Visuospatial Memory Test-Revised (BVMT-R) scores and MRI-derived brain measurements or disability levels. This may be due to the BVMT-R’s primary focus on memory domains, which may be less sensitive to early structural changes than the processing speed evaluated by the SDMT. Our findings support that SDMT is a more sensitive tool for detecting early cognitive dysfunction in MS patients [35,36]. Other studies found that significant improvement in visuospatial memory, as measured by the BVMT-R, was observed in MS patients following corticosteroid treatment, with marked gains evident at the one-month follow-up compared to pretreatment scores. The initial differences between the MS group and healthy controls further support the sensitivity of the BVMT-R in detecting cognitive deficits during an MS relapse [37]. While the full BICAMS battery remains the gold standard for cognitive screening in multiple sclerosis patients, practical limitations in clinical settings may restrict its routine use due to time constraints. Recent research evaluating a large cohort of MS patients (*n* = 1320) demonstrated that a shortened version of BICAMS—specifically the combination of the SDMT and BVMT-R—retains excellent sensitivity (92.7%) and can be used to check for cognitive impairment when there is a time limitation [21].

In this study, we used the MRI analysis tool SynthSeg, which shows promise for brain MRI analysis. It is designed to automatically segment brain structures across a wide range of MRI types and image qualities. Unlike many other methods, SynthSeg does not need retraining for different scan types—it can work directly on unprocessed clinical images [38]. It produces standardized, high-resolution outputs that can be used for further analysis. Newer versions, like SynthSeg-robust and WMH-SynthSeg, improve performance even more, especially in scans with white matter lesions or low image quality [38,39]. This kind of automatic, flexible MRI analysis could be a valuable addition to cognitive testing in MS. In the future, combining SynthSeg results with cognitive scores like SDMT or BVMT-R could help researchers better understand how brain structure relates to cognitive problems in MS.

Regarding neurofilament light chain levels, elevated pNfL was associated with a higher number of relapses within the first five years of the disease and with enlargement of the third and fourth ventricles. However, pNfL levels did not correlate significantly with total brain volume, cognitive performance, or disability scores (EDSS) in this cohort. These results suggest that while pNfL may serve as a marker of acute neuronal injury and disease activity, it may not directly reflect chronic neurodegenerative changes or cognitive decline in the absence of concurrent inflammation [29,40].

These findings are consistent with the recognized limitations of pNfL, including its sensitivity to nonspecific neuronal damage and confounding factors such as age and comorbidities [11,41]. The absence of correlation between pNfL levels and EDSS or cognitive test scores may also be influenced by the cross-sectional design of the study, the relatively small sample size, and the moderate disability range of our patient cohort (median EDSS 2.5).

The structural MRI correlations suggest that EDSS reflects not only spinal cord or motor pathway damage but also global neurodegeneration across grey and white matter compartments. Notably, subcortical grey matter atrophy showed the strongest association with EDSS, which may be related to its involvement in motor, cognitive, and emotional regulation. These data support the use of volumetric MRI and clinical history of relapse as relevant markers in assessing disease progression and guiding treatment decisions [42,43]. There was a statistically significant inverse relationship between cortical grey matter volume (in both hemispheres and overall) and disability levels (as measured by EDSS) in our study sample. Individuals with lower grey matter volume tend to have higher EDSS scores, indicating greater disability. However, the correlation was relatively weak (r = −0.32), and cortical grey matter volume explained only a small portion of the variability in disability. The remaining variation in EDSS scores may be attributed to other factors, such as involvement of other brain regions, spinal cord damage, or non-structural factors like inflammation or patient-specific characteristics.

Our findings demonstrate the volume of the left thalamus exhibited a stronger negative correlation with EDSS, suggesting that thalamic atrophy may be a more sensitive marker of clinical progression. The moderate strength of this association also supports the potential use of thalamic volume as an imaging biomarker for tracking disease progression and evaluating treatment effects in clinical settings. Although the findings show strong statistical reliability, further studies are needed to confirm these results and strengthen the evidence base, as statistical significance does not necessarily imply a direct pathophysiological relationship.

While MRI-based metrics appear more robust in reflecting neurodegenerative progression and cognitive impairment in RRMS, pNfL remains a valuable tool for monitoring inflammatory activity and disease exacerbations [8,44]. Other studies report similar findings, showing that during acute relapses in multiple sclerosis, NfL levels can be up to ten times higher than in patients in remission [45]. Also, they are notified that in RRMS patients NfL levels are much higher than in healthy individuals [29,40] and a higher baseline NfL level predicts brain and spinal cord atrophy, as well as future EDSS progression in RRMS and SPMS [46]. Future studies should incorporate longitudinal designs to better capture the dynamic interplay between inflammatory and neurodegenerative processes and their impact on clinical outcomes.

Despite the inclusion of cognitive assessments and EDSS score evaluations, as well as the consideration of a potential blood biomarker (pNfL), the present study is subject to several limitations that warrant acknowledgment. Primarily, the relatively small sample size and absence of a control group limited the statistical power of the analysis, contributing to findings that may be interpreted as ambiguous. Furthermore, the cohort examined in this study consisted of a demographically homogeneous group of MS patients, characterized by younger age, shorter disease duration, and relatively low EDSS scores. This homogeneity may have constrained the generalizability of the results and the strength of the correlations, potentially limiting the observed range of neurodegeneration. Additionally, the study design did not account for the influence of disease-modifying therapies, which could potentially alter both disease progression and cognitive outcomes. Moreover, the number of regions analyzed increases the risk of Type I error. However, our interpretations focused on correlations with consistent clinical or anatomical relevance (e.g., thalamus, subcortical grey matter).

## 5. Conclusions

This study highlights that MRI-derived grey and white matter volumes are strong indicators of cognitive performance and disability in RRMS, while pNfL levels are more closely associated with early inflammatory activity rather than chronic neurodegeneration. SDMT emerged as a particularly sensitive measure of cognitive impairment linked to brain atrophy, whereas BVMT-R showed less consistent associations.

Although pNfL is a promising biomarker for monitoring disease activity, its utility as a standalone predictor of cognitive or disability progression in RRMS appears limited. Combining MRI volumetric analysis with pNfL measurements may provide a more comprehensive assessment of the state of the disease. Longitudinal studies with larger sample sizes are needed to confirm these findings and clarify the roles of different biomarkers in the complex pathology of multiple sclerosis.

## Figures and Tables

**Table 1:** Demographic and clinical characteristics of the study cohort (*n* = 49).

Variable	Value
Gender, *n* (%)	
Female	25 (51.0%)
Male	24 (49.0%)
Age (years), median [Q1; Q3]	38.0 [30.0; 46.0]
Expanded Disability Status Scale (EDSS), median [Q1; Q3]	2.50 [2.00; 3.50]
Plasma NfL (pg/mL), median [Q1; Q3]	6.30 [4.60; 10.9]
Duration of the disease (years), median [Q1; Q3]	8.00 [6.00; 10.0]
Symbol Digit Modalities Test (SDMT), median [Q1; Q3]	82.3 [74.0; 92.3]
Brief Visuospatial Memory Test-Revised (BVMT-R), median [Q1; Q3]	3.00 [2.00; 3.00]
Education, *n* (%)	
Secondary school	19 (38.8%)
University	30 (61.2%)
Clinical relapses within the first 5 years, median [Q1; Q3]	3.00 [3.00; 4.00]

## Data Availability

The data are available upon request due to ethical restrictions. Requests to access datasets should be directed to elinapolunosika@gmail.com.

## Abbreviations

The following abbreviations are used in this manuscript:

RRMS	Relapsing–remitting multiple sclerosis
SDMT	Symbol Digit Modalities Test
BVMT-R	Brief Visuospatial Memory Test-Revised
pNfL	Plasma neurofilament light chain
NfL	Neurofilament light chain
EDSS	Expanded Disability Status Scale
MRI	Magnetic resonance imaging
SPMS	Secondary progressive multiple sclerosis
BICAMS	Brief International Cognitive Assessment for Multiple Sclerosis
CI	Confidence interval
